# Clinical pharmacist interventions in nutrition-and drug-related problems in critically ill patients with renal dysfunction: a non-randomized controlled study

**DOI:** 10.3389/fmed.2024.1473719

**Published:** 2024-10-25

**Authors:** Betül Özgan, Yunus Emre Ayhan, Sule Apikoglu, Sait Karakurt

**Affiliations:** ^1^Department of Clinical Pharmacy, Marmara University Faculty of Pharmacy, Istanbul, Türkiye; ^2^Department of Clinical Pharmacy, Prof. Dr. Cemil Taşcıoğlu City Hospital, Istanbul, Türkiye; ^3^Department of Pulmonary and Intensive Care Unit, Marmara University Faculty of Medicine, Istanbul, Türkiye

**Keywords:** clinical pharmacist, intensive care unit, renal dysfunction, drug-related problem, nutrition

## Abstract

**Background:**

Critically ill intensive care unit (ICU) patients often face life-threatening drug-related problems (DRPs) and malnutrition. Clinical pharmacists (CPs) play a crucial role in mitigating these issues and improving outcomes.

**Aim:**

This study was designed to detect, prevent, reduce or resolve nutrition-related problems (NRPs) and DRPs in intensive care patients with renal dysfunction through clinical pharmacy services.

**Method:**

This 9-month, prospective, non-randomized, controlled study was conducted in the ICU. During the intervention period (IP), CP recommendations addressing NRPs and DRPs were provided to the healthcare team. NRPs were evaluated using an expert-developed enteral nutrition consensus protocol, while DRPs were classified according to the Pharmaceutical Care Network Europe (PCNE) Classification for Drug-Related Problems Version 9.1.

**Results:**

The study included 60 patients with a median age of 73 years (IQR: 60.5–80). A total of 504 DRPs (8.4 per patient) were identified across all patients. DRPs were decreased by 50% during the IP compared to the observation period (OP) (*p* < 0.001). The most common causes of DRPs were ‘too low a drug dose’ (22.2%), ‘drug–drug interactions’ (17%), and ‘too high a drug dose’ (16.4%). Of the recommendations made to the prescribing physician, 140 (97.9%) were accepted. In the IP, targeted calorie and protein supplementation was fully achieved in more patients (*p* < 0.05). The most common recommendations included ‘changes in the rate of nutrition’ (66.7%), ‘vitamin supplementation’ (16.7%), and ‘changes in enteral nutrition products’ (7.7%).

**Conclusion:**

This study highlights the high incidence of DRPs and malnutrition risk in ICU patients with renal dysfunction, emphasizing the vital role of clinical pharmacists. Their collaboration with healthcare professionals significantly reduced both DRPs and NRPs.

## 1 Introduction

Critically ill patients requiring treatment in the intensive care unit (ICU) often suffer from potentially life-threatening drug-related problems (DRPs). The Pharmaceutical Care Network Europe (PCNE) defines a DRP as “an event or circumstance involving drug therapy that actually or potentially interferes with desired health outcomes” (https://www.pcne.org/working-groups/2/drug-related-problem-classification, Accessed September 10, 2022). Due to the complexity of critical patient care and intricate treatment protocols, the rate of DRPs is higher in the ICU compared to other medical services (1). Renal dysfunction is a risk factor that increases DRP rates in the ICU (2–4). The global incidence of acute kidney injury (AKI) is reported to be 22% (5), while it rises to 57% in the ICU (6). Patients with impaired renal function in the ICU have been shown to require more pharmaceutical interventions than those with normal renal function (7). Involvement of clinical pharmacists (CPs) as pharmacotherapy specialists in routine patient care, in collaboration with other healthcare professionals contributes to improved patient outcomes by reducing DRP rates (8).

In addition to DRPs, the nutritional needs of critically ill patients are complex and vary according to the stage of their illness (9, 10). Malnutrition can occur in critically ill patients at a rate ranging from 38 to 78% (9). It is associated with increased infectious complications, multi-organ dysfunction, prolonged hospitalization, and high mortality (11). Currently, many health centers manage nutritional therapy through multidisciplinary teams, with nutritional support pharmacists playing a vital role in maintaining and improving patients’ optimal nutritional status. The primary function of the pharmacist in the team is to provide nutritional therapy tailored to each patient’s needs (12). This study was designed to assess the effect of clinical pharmacy services in identification, prevention, and resolution of DRPs and nutrition-related problems (NRPs) in intensive care patients with renal dysfunctions.

## 2 Materials and methods

### 2.1 Ethical approval

This study received ethical approval from the Clinical Research Ethics Committee (approval no: 09.202211565, date: 02.09.2022). All procedures adhered to the ethical standards of the University of Siena and the principles of the 1964 Declaration of Helsinki and its subsequent amendments.

### 2.2 Setting and study design

The study was a prospective, observational study conducted in an 8-bed internal ICU of a university hospital between November 1, 2022, and July 21, 2023. The study consisted of two phases: a 4-month observation period (OP) and a 4-month intervention period (IP). There was a 1-month lag-period (LP) between the two phases to allow for the discharge of OP patients from the ICU.

### 2.3 Participants

For the sample size of the study, it was determined that at least 26 patients should be included in each group, based on a calculation using a standard deviation of 1, an alpha level of 0.05, and a power of 95%. This calculation was based on literature indicating that DRP rates could be reduced from 1.96 to 0.94 (approximately 50%) per patient following CP recommendations. A total of 60 patients were included, with at least 30 in each group, taking into account the 15% margin for potential dropout (13).

**Figure 1:**
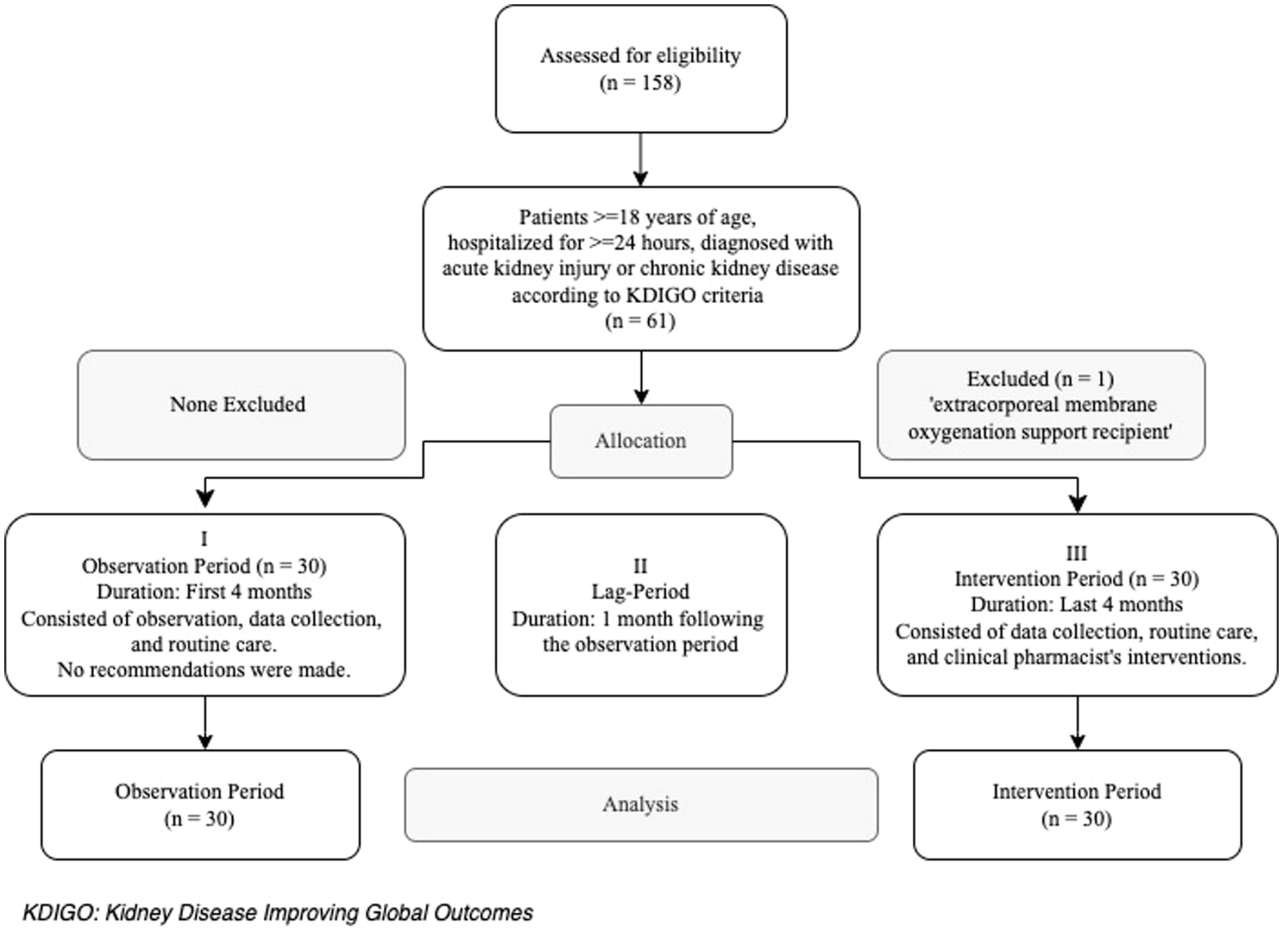
Flow chart of the study.

Inclusion criteria were patients aged ≥18 years, hospitalized for ≥24 h in the ICU and diagnosed with AKI or chronic kidney disease (CKD) according to Kidney Disease Improving Global Outcomes (KDIGO) criteria (14, 15). The exclusion criteria was the receipt of extracorporeal membrane oxygenation support (Figure 1). Patients and/or their surrogate family members were informed about the study and invited to participate. The first 30 patients from each group (OP and IP) who met the inclusion criteria and provided informed consent were included to the study. Both written and verbal consents were obtained from those who accepted to participate.

### 2.4 Study design

This prospective, observational study consisted of two phases: a 4-month observation period (OP) and a 4-month intervention period (IP), with a 1-month lag-period (LP) in between, to allow for the OP patients discharge from the ICU.

Throughout the study, the CP collaborated with the same attending physician, who is a professor of intensive care medicine. During both phases, the CP collected and recorded patients’ demographic and clinical data, including medical history, medications, laboratory values, microbiology culture results, nutritional status, Acute Physiology and Chronic Health Evaluation II (APACHE II) score, Nutrition Risk in Critically ill (NUTRIC) score. On weekdays, the CP participated in routine patient rounds, regularly reviewing physician orders and patients to identify of any manifest and/or potential DRPs and NRPs. Patients were followed until discharge or death.

During the OP, patients received routine ICU care. The CP conducted medication and nutrition review to identify DRPs and NRPs, but did not implement any interventions. Routine ICU care included dietician consultations and general visits once weekly.

During the LP, the CP reviewed the most common DRPs and NRPs encountered during the OP and provided face-to-face training to the healthcare team, which consisted of the attending physician, ICU specialists, and resident physicians. This training covered the importance, prevention, and resolution of these problems. The ICU healthcare team was also informed about the enteral nutrition (EN) protocol that was developed by the expert team. These training sessions were repeated whenever new residents joined the team and as needed.

During the IP, besides the routine ICU care, the CP continued to provide medication and nutrition reviews for DRPs and NRPs and made recommendations to the attending physician, regarding the identified issues.

### 2.5 Main outcome measures

The primary outcomes of the study included the frequencies of DRPs, the number of DRPs prevented or resolved during the IP, and assessment of nutritional parameters (e.g., NUTRIC score, protein and calorie intake in the first 7 days and after the 7th day, time to reach target nutrition, complications, and vitamin supplements) under the protocol. Secondary outcomes included a description of medication groups involved in DRPs and identification of DRP risk factors.

### 2.6 Identification of DRPs

DRPs were classified using the Turkish version of the PCNE Classification for Drug-Related Problems V9.1 (https://www.pcne.org/working-groups/2/drug-related-problem-classification, Accessed September 10, 2022). UpToDate^®^ and Micromedex^®^ database were utilized to provide information on indications, contraindications, dosages (considering renal impairment, hepatic impairment, older adults and obesity), administration, adverse reactions, monitoring parameters, and pharmacology of the drugs. The Sanford Guide to Antimicrobial Therapy was also used for information on antimicrobial drugs.

Potential drug–drug interactions (pDDIs) were identified using the UpToDate^®^ database. Major and contraindicated interactions were recorded as DRPs, with clinical significance were determined collaboratively by the attending physician and the CP. ‘Not clinically significant’ pDDIs were defined as at least one of the following: ‘interactions that could not be avoided in the ICU and/or the stated risk does not apply to the patient and/or no change in treatment or administration modality is required’. No recommendation was made regarding ‘not clinically significant’ pDDIs.

### 2.7 Identification of NRPs

In this study, only EN-related problems were assessed, as the patient population rarely received parenteral nutrition. The attending physician and the CP defined the NRPs as ‘errors or complications related to EN therapy, such as issues with timing, method, rate of administration, and choice of nutritional product, which could prevent the achievement of desired nutritional goals’.

The evaluation of NRPs in critically ill patients with renal dysfunction utilized an EN protocol (Supplementary Table S1) developed from the most current guidelines from the European Society for Clinical Nutrition and Metabolism (ESPEN) and the American Society for Parenteral and Enteral Nutrition (ASPEN) (16–19). This protocol was created through consensus among an expert team, consisting of two clinical pharmacy specialists, an intensive care medicine specialist, and an assistant clinical pharmacist.

Nutritional status was assessed using parameters such as the NUTRIC score, EN duration, achievement of calorie and protein goals in the first 7 days, and after the 7th day, as well as the time taken to reach these goals.

### 2.8 Clinical pharmacist interventions

Recommendations made by the CP to address the identified DRPs and NRPs included adding or stopping medications, changing to alternative treatments, changing routes of administration, dose adjustments, side effect management, therapeutic drug monitoring, or optimizing drug administration techniques. Recommendations were directed only to the prescribing physician.

A problem where the CP was consulted or intervened before a drug was prescribed, was recorded as a “prevented DRP”; a problem where the CP intervened before a prescribed drug was administered was recorded as a “potential DRP”; and a problem where the CP intervened after a drug was administered was recorded as a “manifest DRP”.

### 2.9 Statistical analysis

Statistical analyses were performed using IBM SPSS Statistics for Windows, Version 29.0 (Armonk, New York: IBM Corp.). Continuous variables were expressed as median (interquartile range [IQR]), and nominal and ordinal variables were expressed as *n* (%). Normality of continuous variables was assessed using the Kolmogorov–Smirnov test. Differences between two groups for the non-normally distributed data were compared using the nonparametric Mann–Whitney U test. Chi-square tests were used to analyze the relationships between categorical data. The risk status of different clinical conditions for DRPs was determined by odds ratio (OR). All data are presented within 95% confidence intervals, and a *p*-value <0.05 was considered as statistically significant.

## 3 Results

Out of the 158 patients hospitalized in the internal ICU during the OP and IP, 60 patients were included in the study (30 in the OP and 30 in the IP) (Figure 1). More than half of the patients were female, and the median (IQR) age was 73 (60.5–80) years. The most common comorbidities were hypertension (55%) and diabetes (46.7%), while the mortality rate was 55%. The prevalence of comorbidities other than CKD was similar in both groups (*p* > 0.05); however, the prevalence of CKD was higher in the OP patients compared to the IP patients (46.7% vs. 6.7%, respectively; *p* = 0.001). The APACHE II score was higher in the IP patients compared to the OP patients (24 vs. 29, *p* = 0.003). The most common reason for ICU admission was respiratory disorders in the IP (43.3%), while it was infection (20%) and neurologic disorders (20%) in the OP (*p* = 0.025). Of all patients, 27% had a diagnosis of CKD, 73% had developed AKI, and 55% received EN (Table 1).

**Table 1:** Demographic and general clinical characteristics of the patients.

	OP (*n* = 30) median (IQR)	IP (*n* = 30) median (IQR)	Total (*n* = 60) median (IQR)	*p*
Age	72.5 (55.2–80)	73 (62.25–81.5)	73 (60.5–80)	0.673
Body mass index (kg/m^2^)	24.65 (22.63–28)	29.1 (25.4–32.9)	26 (26.95–30.25)	**0.017**
Charlson Comorbidity Index	5 (3–7)	5 (3.75–7)	5 (3.25–7)	0.952
APACHE II	24 (16–26.5)	29 (24.5–32)	25.5 (18.25–30)	**0.003**
Number of comorbidities	4 (3–6)	3.5 (2–5)	4 (2.25–5)	0.281
Length of hospital stay (days)	26 (10.5–50.75)	25.5 (10–57.5)	25.5 (10.25–53)	0.923
Length of ICU stay (days)	11.5 (6.25–26)	12 (5.5–27.5)	12 (6.25–26)	0.882
Length of follow-up (days)	11.50 (6–18.25)	8.5 (4–25)	10.5 (6–21.75)	0.706
Number of drugs on admission	8 (7–10)	7.5 (6–10)	8 (6.25–10)	0.367
Number of drugs on discharge	8.5 (6.75–12)	10 (6–12)	9 (6–12)	0.906
Number of drugs during follow-up	10 (8–12)	9 (7–11)	9 (7.25–11)	0.384
Basal serum creatinine (mg/dL)	0.86 (0.75–2.55)	0.8 (0.7–0.79)	0.8 (0.7–1.17)	**0.025**
Basal eGFR (mL/min/1.73 m^2^)	61 (22.5–89.5)	86.5 (67.5–95.5)	78 (60–95)	**0.032**
Duration with RRT (days)	6 (2.25–4.65)	10 (3.5–10)	10 (3–16.5)	0.367
Duration with MV (days)	9.5 (3.5–7.9)	12 (5.25–9.70)	10 (4.5–26.25)	0.307
Duration with AKI (days)	9.5 (5–11.75)	6.5 (3.25–18)	8.5 (4–13)	0.893
Duration with CKD (years)	5 (2–6)	10	5 (2–6.75)	0.143

	*n*	%	*n*	%	*n*	%	*p* value
Sex							0.6
Male	14	46.7	11	36.7	25	41.7	
Female	16	53.3	19	63.3	35	58.3	
Admission diagnosis							
Respiratory disorder	13	43.3	5	16.7	18	30	**0.025**
Sepsis/septic shock	7	23.3	4	13.3	11	18.3	
Infection	3	10	6	20	9	15	
Renal disorder	3	10	0	0	3	5	
Neurological disorder	3	10	6	20	9	15	
Postoperative care	1	3.3	5	16.7	6	10	
Endocrine disorder	0	0	2	6.7	2	3.3	
Bleeding	0	0	2	6.7	2	3.3	
Comorbidities							
Hypertension	17	56.7	16	53.3	33	55	1
Diabetes mellitus	16	53.3	12	40	28	46.7	0.44
Chronic kidney disease	14	46.7	2	6.7	16	26.7	**0.001**
Coronary artery disease	11	36.7	8	26.7	19	31.7	0.58
COPD	8	26.7	3	10	11	18.3	0.18
Cancer	7	23.3	11	36.7	18	30	0.4
Cerebrovascular disorder	7	23.3	4	13.3	11	18.3	0.5
Atrial fibrillation	6	20	7	23.3	13	21.7	1
Heart failure	6	20	4	13.3	10	16.7	0.73
Others	29		34		63		
Renal replacement therapy	16	53.3	13	43.3	29	48.3	0.6
Mechanical ventilation	20	66.7	22	73.3	42	70	0.12
Nutrition							
IV dextrose	2	6.7	3	10	5	8.3	0.369
Oral	13	43.3	8	26.7	21	35	
Enteral	14	46.7	19	63.3	33	55	
Total parenteral nutrition	1	3.3	0	0	1	1.7	
ICU mortality	13	43.3	20	66.7	33	55	0.12
Stage of AKI							
Stage 1	7	43.8	5	17.9	12	27.3	0.179
Stage 2	2	12.5	5	17.9	7	15.9	
Stage 3	7	43.8	18	64.3	25	56.8	
Stage of CKD							
G2	2	14.3	0	0	2	12.5	0.797
G3b	3	21.4	1	50	4	25	
G4	1	7.1	0	0	1	6.3	
G5	8	57.1	1	50	9	56.3	

AKI: Acute Kidney Injury; APACHE II: The Acute Physiology and Chronic Health Evaluation II; CKD: Chronic Kidney Disease; COPD: Chronic Obstructive Pulmonary Disease; eGFR: estimated Glomerular Filtration Rate; ICU: Intensive Care Unit; IP: Intervention Period; IQR: Interquartile Range; MV: Mechanical Ventilation; OP: Observation Period; RRT: Renal Replacement Therapy; bold values indicate statistical significance.

A total of 504 DRPs were identified for all patients (8.4 DRPs per patient). The majority (98.3%) of patients had at least one DRP, with the number of DRPs per patient being 11.2 in the OP and 5.6 in the IP (*p* < 0.001). The most common causes of DRPs were “too low a drug dose” (C3.1; 22.2%), “an inappropriate combination of drugs with other drugs” (C1.3; 17%), and “too high a dose of a single active ingredient” (C3.2; 16.4%). In the IP, compared to the OP, the rates of DRP causes decreased significantly: by 67% for “no indication for the drug”, by 47% for “inappropriate combination of drugs with other drugs”, by 67% for “no or incomplete drug treatment despite existing indication”, by 45% for “low drug dose”, by 53% for “high drug dose”, by 67% for “too frequent dosing regimen”, by 100% for “not available prescribed drug”, and by 69% for “incorrect administration time” (coded as other reason) (*p* < 0.05, for all) (Table 2).

**Table 2:** Classification of identified drug-related problems according to the PCNE classification for drug-related problems version 9.1.

Causes	OP *n* (%)	IP *n* (%)	Total *n* (%)	*p*
**1. Drug selection**	**115 (34.7)**	**51 (31)**	**166 (33.5)**	
C1.1. Inappropriate drug according to guidelines/formulary	13 (3.9)	5 (3)	18 (3.6)	0.106
C1.2. No indication for drug	12 (3.6)	4 (2.4)	16 (3.2)	**0.020**
C1.3. Inappropriate combination of drugs, or drugs and herbal medications, or drugs and dietary supplements	55 (16.6)	29 (17.7)	84 (17)	**0.018**
C1.4. Inappropriate duplication of therapeutic group or active ingredient	5 (1.5)	3 (1.8)	8 (1.6)	0.451
C1.5. No or incomplete drug treatment in spite of existing indication	30 (9)	10 (6.1)	40 (8.1)	**0.006**
**2. Drug form**	**33 (10)**	**24 (14.6)**	**57 (11.5)**	
C2.1. Inappropriate drug form/formulation (for this patient)	33 (10)	24 (14.6)	57 (11.5)	0.148
**3. Dose selection**	**149 (45)**	**80 (48.8)**	**229 (46)**	
C3.1. Drug dose too low	71 (21.5)	39 (23.8)	110 (22.2)	**0.007**
C3.2. Drug dose of a single active ingredient too high	55 (16.6)	26 (15.9)	81 (16.4)	**0.007**
C3.3. Dosage regimen not frequent enough	4 (1.2)	5 (3)	9 (1.8)	0.490
C3.4. Dosage regimen too frequent	12 (3.6)	4 (2.4)	16 (3.2)	**0.035**
C3.5. Dose timing instructions wrong, unclear or missing	7 (2.1)	6 (3.7)	13 (2.6)	0.949
**4. Treatment duration**	**4 (1.2)**	**1 (0.6)**	**5 (1)**	
C4.2. Duration of treatment too long	4 (1.2)	1 (0.6)	5 (1)	0.165
**5. Dispensing**	**4 (1.2)**	**0 (0)**	**4 (0.8)**	**0.040**
C5.1. Prescribed drug not available	4 (1.2)	0 (0)	4 (0.8)	
**9. Other**	**26 (7.9)**	**8 (4.8)**	**34 (6.9)**	**0.005**
C9.2. Other cause; specify	26 (7.9)	8 (4.8)	34 (6.9)	
**Total drug related problems**	335	169	504	**<0.001**

IP: Intervention Period; OP: Observation Period; PCNE: Pharmaceutical Care Network Europe; bold values indicate statistical significance.

**Figure 2:**
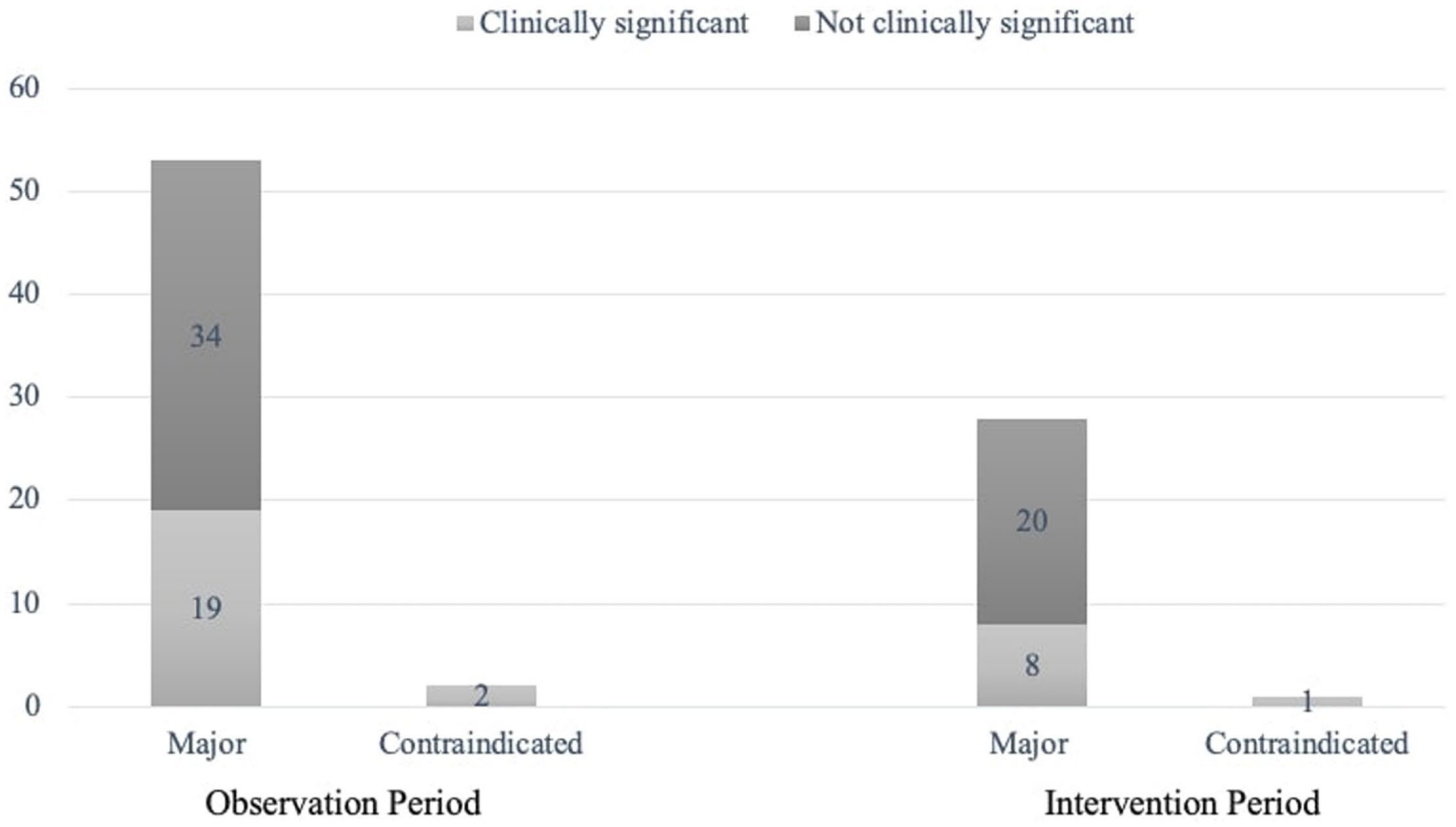
Potential drug–drug interactions.

Intervention recommendations was proposed for the majority (84.6%, *n* = 143/169) of the DRPs identified in the IP. Most (88.5%, *n* = 23/26) of the DRPs for which intervention was not recommended, were related to “inappropriate combination of drugs with other drugs”. The frequency and clinical significance of pDDIs identified in both groups are shown in Figure 2. Of the recommendations made to the prescribing physician, 140 (97.9%) were accepted, and 87 DRPs were prevented and classified as “prevented DRPs.” In this study, 95.7% (134/140) of the accepted recommendations were fully implemented, two were partially implemented, and four were omitted.

When both periods were analyzed together, antimicrobial drugs (33.2%), nervous system drugs (21.4%), and digestive system and metabolism drugs (14.7%) were the most common groups associated with DRPs. The drugs most frequently involved in DRPs were enoxaparin (9.1%), meropenem (8.2%), vancomycin (7.7%), pantoprazole (6%), and piperacillin-tazobactam (5.2%). When pDDIs were analysed across both periods, the drugs most frequently involved in interactions were enoxaparin (14%), dexmedetomidine (12%), acetylsalicylic acid (7.6%), tramadol (6.4%), levetiracetam (5.7%), and valproic acid (5.7%).

In the IP, targeted calorie and protein supplementation was fully achieved in more patients. Significant increases were observed in the percentages of patients reaching protein target in the first 7 days (*p* = 0.008), and calorie and protein targets after the 7th day (*p* = 0.007 and *p* < 0.001, respectively). Similarly, the amount of protein provided in the first 7 days (*p* = 0.034) and the amounts of calories and protein provided after the 7th day were significantly higher in IP compared to OP (*p* = 0.043 and *p* < 0.001, respectively). Serum vitamin levels (vitamin D, vitamin B12, folic acid) were measured in more patients in the IP (*n* = 13, 15, 16, respectively) compared to the OP (*n* = 1, 2, 2, respectively) and patients with low results were provided with the necessary supplements. Nutrition-related complications developed in 26.4% of the patients receiving EN, with the most common complications being diarrhea (12.1%) and vomiting (9.1%) (Table 3). A total of 78 recommendations were made to the ICU team to optimize clinical nutrition, and 96.2% of these recommendations were accepted. These recommendations included initiation of nutrition, changes in the rate of nutrition, addition or discontinuation of prokinetic agents, changes in EN products, and vitamin supplementation (Table 4).

**Table 3:** Nutritional characteristics of the patients.

	OP, median (IQR)	IP, median (IQR)	Total, median (IQR)	*p*
NUTRIC	6.5 (5–7.25)	7 (3–8)	7 (5–7.5)	0.640
Enteral nutrition duration (days)	15 (8–22.25)	14 (6–25)	15 (7–23)	0.855
Achieving the calorie goal in the first 7 days (%)	25 (0–60)	75 (29.5–93)	53.5 (10.3–86)	0.08
Calorie intake for the first 7 days (kcal/day)	544 (387–810)	823 (521–1,122)	595 (475.25–900.25)	**0.034**
Achieving the protein goal in the first 7 days (%)	0 (0–25)	57 (0–73)	12.5 (0–57)	**0.008**
Protein intake for the first 7 days (g/day)	31 (22.7–48)	52.7 (30–69.65)	39.9 (25.2–59.88)	0.099
Time to reach target nutrition (days)	16	6 (4–20)	6 (4–19.5)	0.529
Achieving the calorie goal after the 7th day (%)	0 (0–22)	59.3 (29–100)	31 (0–68.3)	**0.007**
Calorie intake after the 7th day (kcal/day)	827 (616–1,120)	1,124 (1080–1,440)	1,095 (773.5–1280.25)	**0.043**
Achieving the protein goal after the 7th day (%)	0	59.3 (29–88.9)	26.25 (0–67.3)	**<0.001**
Protein intake after the 7th day (g/day)	43.4 (27.5–53.5)	75.6 (56.8–86.2)	55.3 (41.9–76.2)	**<0.001**

	*n*	%	*n*	%	*n*	%	*p*
Enteral nutrition route							
Nasogastric tube	11	78.6	17	89.5	28	84.8	
Orogastric tube	1	7.1	0	0	1	3	0.129
Percutaneous endoscopic gastrostomy	0	0	2	10.5	2	6.1	
Percutaneous endoscopic jejunostomy	2	14.3	0	0	2	6.1	
Complication							
Diarrhea	2	14.3	1	5.3	3	9.1	0.361
Vomiting	1	7.1	1	5.3	2	6.1	
Diarrhea and vomiting	0	0	1	5.3	1	3	
No complication	11	78.6	16	84.1	27	81.8	
Was a prokinetic agent used?							
Yes	3	21.4	6	31.6	9	27.3	0.698
No	11	78.6	13	68.4	24	72.7	
Has nutrition been interrupted?							
Yes	11	78.6	8	42.1	19	57.6	**0.036**
No	3	21.4	11	57.9	14	42.4	

IP: intervention period; IQR: interquartile range; NUTRIC: the nutrition risk in critically ill; OP: observation period; bold values indicate statistical significance.

**Table 4:** Enteral nutrition interventions.

Interventions	*n*	%	Acceptance of the intervention (%)
Initiation of nutrition	3	3.9	100
Changes in enteral nutrition product	6	7.7	100
Changes in the rate of nutrition	52	66.7	94
Initiation of a prokinetic agent	1	1.3	100
Discontinuation of a prokinetic agent	3	3.8	100
Vitamin supplement	13	16.7	100

The risk factors that increase the number of DRPs for all periods were analyzed, and the risk ratios are presented in Table 5. DRP risk factors were identified as CKD, duration of antibiotic treatment, length of follow-up, number of comorbidities, length of hospital stay, and number of drugs at ICU admission (*p* < 0.05 for all), with CKD having the highest OR (CI) [4.253 (1.247–14.511)] among all.

**Table 5:** Risk factors for drug-related problems.

Risk factors	OR (confidence interval)	*p* value
Mechanical ventilation support	3.850 (1.086–13.647)	0.331
Chronic kidney disease	4.253 (1.247–14.511)	0.017
Number of comorbidities	1.386 (1.036–1.854)	0.028
Renal replacement therapy	1.948 (0.692–5.485)	0.205
Nutrition support	2.531 (0.867–7.387)	0.086
Length of intensive care unit stay	1.026 (0.995–1.057)	0.096
Length of follow-up	1.077 (1.022–1.134)	0.006
Length of hospital stay	1.021 (1.001–1.041)	0.043
Duration with renal replacement therapy	1.058 (0.977–1.145)	0.163
Duration with mechanical ventilation	1.005 (0.973–1.039)	0.748
Duration with acute kidney injury	1.065 (0.987–1.150)	0.105
Number of drugs at intensive care unit admission	1.334 (1.047–1.700)	0.020
Number of drugs on discharge	1.153 (0.999–1.331)	0.051
Duration with antibiotic treatment	1.115 (1.037–1.198)	0.003

## 4 Discussion

In our study, we evaluated the effect of clinical pharmacy services in identification, prevention, and resolution of DRPs and NRPs in intensive care patients with renal dysfunctions.

The reported DRP rate per patient in ICU studies ranges from 0.83 to 7.26 (3, 4, 20–23). The incidence of DRPs in patients with renal dysfunction is 1.63 to 9 times higher than in those with normal renal function (4, 7, 20, 23). Our study identified a higher number of DRPs than reported in the literature, reflecting our patient population’s renal dysfunction. We observed a significant decrease in the DRP rate per patient during the IP with CP involvement. While some controlled ICU studies noted a significant reduction in DRPs following CP intervention, others found no substantial differences between periods (4, 24–26). Variability in results may stem from factors such as differences in study settings, team composition changes, inadequate training, or prior experiences with CP.

In our study, dose selection emerged as the most frequent issue. This aligns with findings from Albayrak et al. and Chiang et al. where 30.5 and 30.9% of patients had renal dysfunction, respectively and dose selection was also a prevalent concern affecting 54.4 and 55.8% of patients, respectively (3, 7). Jiang et al. reported inappropriate drug frequency and dosing isuues in 37% of patients, with 74.7% of those with renal dysfunction or on renal replacement therapy (RRT) affected (20). In studies with lower renal dysfunction rates, drug selection was the predominant problem (22–60%) (4, 22, 23). These findings highlight the lack of awareness regarding necessary adjustments in drug dosing and frequency based on changing drug excretion rates, underscoring the importance of including CP in the multidisciplinary team to ensure proper dosing in patients with renal dysfunction.

We also evaluated the clinical significance of contraindicated and major interactions with input from both the CP and the ICU physician. Only 29 (34.5%) of the 84 pDDIs recorded necessitated changes in drug therapy. This emphasizes the need for evaluating drug interaction data on a patient-specific basis in collaboration with the CP. The total and clinically significant pDDI were higher in the IP (*p* = 0.024). This suggests that CP involvement in checking drug interactions prior to prescribing new medications effectively reduced the number of pDDIs. Aghili and Kasturirangan reported a similar rate of interactions requiring treatment adjustments at 31% (27). A systematic review indicated that 58% of ICU patients experience at least one pDDI, with not all interactions being clinically significant (28). Our study identified enoxaparin, dexmedetomidine, aspirin, tramadol, levetiracetam, and valproic acid as the most frequently involved drugs in interactions. In a systematic review and meta-analysis of 39 ICU studies, drugs most frequently involved in interactions were identified as aspirin, insulin, clopidogrel, furosemide, and omeprazole (28). Variability in reported drug interactions can be attributed to differences in population characteristics, treatment protocols, logistical issues, and the timing of studies.

Literature reports varying acceptance rates for CP recommendations, with figures ranging from 95 to 99.2%, similar to our findings, while others reported lower acceptance rates (67.3–93%) (3, 4, 7, 20–23, 26, 29, 30). High acceptance rates obsereved in this study may reflect the impact of a previous study evaluating clinical pharmacy services within the same ICU, and may be due to the fact that the recommendations were timely and tailored to patient needs.

Our findings, consistent with numerous studies, indicate that antimicrobial drugs frequently contribute to DRPs (22–81%) (7, 20, 22, 23, 30). Other drug categories commonly associated with DRPs included nervous system drugs (32.2%), gastrointestinal system drugs (18.7–27.4%), and antithrombotics (9.6–13.1%) (4,2 8). It is not surprising that the most commonly utilized drug groups in the ICU also represent a significant portion of DRPs (31, 32).

Many studies have explored factors contributing to DRPs and identified patient groups that should be prioritized by the CP. The risk factors identified in Table 5 align with those reported in other ICU studies (4, 21, 23, 25). Notably, CKD significantly increased the number of DRPs. However, the higher CKD prevalence in the OP patients compared to the IP patients (46.7% vs. 6.7%, respectively; *p* = 0.001) and the 50% drop in the frequency of DRPs in the IP compared to the OP may have influenced this outcome. Body mass index, which was significantly higher in the IP patients was not found to impact DRP presence (*p* = 0.426).

Evaluation of patients’ nutritional characteristics revealed high NUTRIC scores, indicating that many were at significant risk of malnutrition and are likely to benefit from aggressive nutritional therapy to improve mortality outcomes. Similar to the literature, the daily calorie and protein intake during the OP was much lower than targets. Contributing factors may include a greater focus on medical treatment, insufficient nutrition monitoring, and a lack of systemic management of nutritional support by the nutritional support team (33, 34). With highly accepted CP recommendations, more patients in IP transitioned to targeted nutrition, resulting in significant increases in the percentage achieving target calorie and protein support. It has been shown in the literature that establishing a nutritional support team, inclusive of pharmacists, or implementing a nutritional protocol leads to improved outcomes in reaching target calorie and protein values, and shortened timelines for nutrition initiation (35, 36). In our study center, the absence of protocol-based nutritional follow-up accentuates the necessity for CP involvement in a high-risk environment for malnutrition, highlighting its role in enhancing critical patient care.

### 4.1 Strengths and weaknesses

This study is significant as it focuses on patients with renal dysfunction, a population where DRPs are prevalent in ICUs in our country. It categorizes DRPs and NRPs while evaluating the effects of CP services. To our knowledge, no similar study exists in our country.

However, our study has limitations, including its conduct in a single center with a relatively small sample size and the lack of evaluation of parenteral nutrition, which was administered to very few patients.

### 4.2 Further research

Future randomized controlled trials will be conducted under CP leadership, and current consensus reports will be established across different disciplines for critically ill patient populations. This will further elucidate the importance of CP.

## 5 Conclusion

In summary, this study examines the impact of CP on evidence-based services for ICU patients with renal dysfunction. The healthcare team highly accepted and implemented recommendations for identifying, resolving, and preventing NRPs and DRPs. The involvement of CP in the ICU team, participating in rounds with other healthcare professionals, significantly reduces DRP frequency and enhances the achievement of nutritional goals.

## Data Availability

The original contributions presented in the study are included in the article/Supplementary material, further inquiries can be directed to the corresponding author.
