# Supplementary material for: Clinical pharmacist interventions in nutrition-and drug-related problems in critically ill patients with renal dysfunction: a non-randomized controlled study
Source: Front Med (Lausanne). 2024 Oct 25;11:1473719. doi: 10.3389/fmed.2024.1473719 (PMC11543425; doi:10.3389/fmed.2024.1473719)
Supplement: Supplementary file 1 [file Table_1.DOCX]

| Supplementary 1. Enteral nutrition consensus protocol [16-19] |
| --- |
| Medical nutrition therapy shall be considered for all patients staying in the ICU, mainly for more than 48 h. |
| A general clinical assessment should be performed to assess malnutrition in the ICU, until a specific tool has been validated. |
| Medical nutrition therapy may be considered for any patient with AKI/AKD, AKI on CKD, CKD with or without KF requiring hospitalization. |
| Oral diet shall be preferred over EN or PN in critically ill patients who are able to eat. |
| If oral intake is not possible, early EN (within 48 h) in critically ill adult patients should be performed/initiated rather than delaying EN. |
| If oral intake is not possible, early EN (within 48 h) shall be performed/initiated in critically ill adult patients rather than early PN. |
| In case of contraindications to oral and EN, PN should be implemented within 3 to 7 days. |
| To avoid overfeeding, early full EN and PN shall not be used in critically ill patients but shall be prescribed within 3 to 7 days. |
| Continuous rather than bolus EN should be used. |
| Gastric access should be used as the standard approach to initiate EN. |
| In patients who do not tolerate full dose EN during the first week in the ICU, the safety and benefits of initiating PN should be weighed on a case-by-case basis. |
| PN should not be started until all strategies to maximize EN tolerance have been attempted. |
| In patients with gastric feeding intolerance not solved with prokinetic agents, postpyloric feeding should be used. |
| In critically ill patients with gastric feeding intolerance, intravenous erythromycin should be used as a first line prokinetic therapy. Alternatively, intravenous metoclopramide or a combination of metoclopramide and erythromycin can be used as a prokinetic therapy. |
| The effectiveness of erythromycin or other prokinetics decreases to one-third after 72 hours and should be discontinued after 3 days. |
| The calorie recommendation of 20–30 kcal/kg/d for ICU patients with AKI reasonably covers average energy needs and can be used when indirect calorimetry is not available. |
| The energy target is 12-25 kcal/kg/d (hypocaloric) in the acute and early stages of the disease, 3-7. The aim should be to provide 25-30 kcal/kg/d (isocaloric) energy after the day. |
| For patients undergoing RRT, the total energy provision by additional calories given in the form of citrate, lactate, and glucose from dialysis/hemofiltration solutions should be included in the calculations to determine the total daily energy provision to avoid overfeeding. |
| The following protein intakes may be prescribed:  Hospitalized patient with AKI, AKI on CKD, CKD, with acute/ critical illness, not on RRT: start with 1 g/kg/day, and gradually increase up to 1.3 g/kg/day if tolerated  Critically ill patients with AKI or AKI on CKD or CKD with kidney failure on conventional intermittent RRT: 1.3-1.5 g/kg/d  Critically ill patients with AKI or AKI on CKD or CKD with kidney failure on CRRT or PIRRT: 1.5 g/kg/d up to 1.7 g/kg/d |
| To ensure the most accurate dosing in clinical practice, the most appropriate EN or PN formula should be chosen based on calories and protein ratio. |
| EN should be delayed;  If shock is uncontrolled and hemodynamic and tissue perfusion goals are not reached, whereas low dose EN can be started as soon as shock is controlled with fluids and vasopressors/inotropes, while remaining vigilant for signs of bowel ischemia  In case of uncontrolled life-threatening hypoxemia, hypercapnia or acidosis, whereas EN can be started in patients with stable hypoxemia, and compensated or permissive hypercapnia and acidosis  In patients suffering from active upper GI bleeding, whereas EN can be started when the bleeding has stopped and no signs of rebleeding are observed  In patients with overt bowel ischemia  In patients with high-output intestinal fistula if reliable feeding access distal to the fistula is not achievable  In patients with abdominal compartment syndrome  If gastric aspirate volume is above 500 ml/6 h |
| Blood glucose should be measured initially (after ICU admission or after artificial nutrition initiation) and at least every 4 h, for the first two days in general. |
| Electrolytes (potassium, magnesium, phosphate) should be measured at least once daily for the first week. |
| In patients with refeeding hypophosphatemia (<0.65 mmol/l or a drop of>0.16 mmol/l), electrolytes should be measured 2-3 times a day and supplemented if needed. |
| Dialysis solutions containing potassium, phosphate, and magnesium should be used to prevent electrolyte disorders during RRT. |
| Because of increased requirements during kidney failure and critical illness, and large effluent losses during RRT, water-soluble vitamins should be monitored and supplemented. Special attention should be given to vitamin C, folate, and thiamine. |
| In patients with severe malnutrition, early PN can be provided instead of not feeding in case of contraindications to EN. |
| In patients deemed to be at high risk for aspiration, post-pyloric, mainly jejunal feeding can be performed. |
| In selected patients with electrolyte and fluid imbalances, concentrated “renal” EN or PN formulas with lower electrolyte content may be preferred over standard formulas. |
| In critically ill patients with measured low plasma levels (25-hydroxy-vitamin D<12.5 ng/ml, or 50 nmol/l) vitamin D3 can be supplemented. In critically ill patients with measured low plasma levels (25-hydroxy-vitamin D<12.5 ng/ml, or 50 nmol/l) a high dose of vitamin D3 (500,000 UI) as a single dose can be administered within a week after admission. |
| If indirect calorimetry is not available, energy intake can be based on adjusted body weight. |
| *AKI: Acute Kidney Injury; AKI: Acute Kidney Injury; CKD: Chronic Kidney Disease; EN: Enteral Nutrition; ICU: Intensive Care Unit; PN: Parenteral Nutrition; RRT: Renal Replacement Therapy* |
